# A simple and rapid evaluation of boar sperm quality using the resazurin colorimetric assay

**DOI:** 10.1590/1984-3143-AR2024-0005

**Published:** 2025-01-20

**Authors:** Fung-Hsiang Chu, Yu-Hsin Chen, Ting-Chieh Kang, Lih-Ren Chen, Hsiu-Lien Herbie Lin

**Affiliations:** 1 Genetics and Physiology Division, Taiwan Livestock Research Institute, Ministry of Agriculture, Tainan, Taiwan; 2 Livestock Management Division, Taiwan Livestock Research Institute, Ministry of Agriculture, Tainan, Taiwan; 3 Southern Region Branch, Taiwan Livestock Research Institute, Ministry of Agriculture, Pingtung, Taiwan

**Keywords:** boar, computer-assisted sperm analysis, flow cytometry, resazurin, resorufin, sperm

## Abstract

Ensuring boar sperm quality before insemination is crucial for maximizing field fertility and efficient pig production. The computer-assisted sperm analysis (CASA) and fluorescence probes combined with flow cytometry (FC) are commonly used techniques for evaluating sperm kinematics and functions, closely related to animal fertility. However, their high cost and complex operations make it challenging to apply them in laboratories or pig breeding farms with limited resources. Here, our aim was to develop a new protocol using a resazurin redox dye to assess boar sperm quality for practical application. We first created simulated semen samples with different levels of sperm quality (0, 20, 40, 60, 80, 100%) at concentrations of 300 and 150 × 10^6^ cells/mL. Subsequently, the simulated semen was used to establish an optimal standard protocol based on the results of the resazurin colorimetric assay. Finally, the condition that showed the strongest correlation between resazurin redox rate and sperm parameters was selected to perform a linear regression test. Two optimal working conditions were identified, involved incubating 10 µL of resazurin reagent with 100 µL of semen for either 20 or 40 min, depending on the sperm concentration of either 300 or 150 × 10^6^ cells/mL. We subsequently conducted a linear regression analysis using data that included the resazurin reaction rate and measurements of sperm parameters. Finally, we obtained two sets of five equations, allowing directly convert the absorbance of the resazurin assay into values for sperm quality parameters. These parameters include total motility, progressive motility, viability, acrosome integrity, and mitochondrial activity. This new protocol is valuable for sperm evaluation because it is cost-effective, time-efficient, and labor-saving.

## Introduction

Pork production plays an important role in providing dietary energy, protein, as well as macro and micronutrients in the human food chain (Datlow et al., 2023). The global pork production is increasing and reached 124.6 million tons in 2022, expanding by 1.8% from 2021 (FAO, 2022). New technologies are constantly being introduced to make pig production more efficient, and assisted reproductive technology (ART) is one of them. Artificial insemination (AI) has been a standard practice in the global pig industry for over fifty years (Knox, 2016). For ensuring the success of AI, it is necessary to guarantee the quality of sperm for field fertility (Tsakmakidis et al., 2010), which depends on accurate and consistent techniques for evaluating semen in vitro (Maside et al., 2023).

The most common in vitro parameters used for predicting boar fertility include sperm concentration, motility, and viability (Namuncura et al., 2020). These parameters can be assessed through conventional microscopy analysis (Dias et al., 2019). More recently, computer-assisted sperm analysis (CASA) and flow cytometry (FC) have been introduced as high-throughput, objective, and precise methods for evaluating sperm kinematics and functionality (Boe-Hansen and Satake, 2019; Valverde et al., 2020). The data collected from CASA or FC were found to be highly correlated with animal fertility (Jung et al., 2015). Additionally, they provide useful information for culling poor-quality samples (Gillan et al., 2005; Mocé and Graham, 2008). Despite CASA and FC both demonstrating competitive accuracy and efficiency for sperm analysis, the expensive equipment and complex manipulations make them difficult to afford or conduct in every spermatology laboratory or breeding field. Therefore, an economical and simple method that overcomes these disadvantages may be valuable as a new approach to assess sperm fertilizing capacity.

The resazurin chemical compound (7-hydroxy-3H-phenoxazin-3-one 10-oxide) is a cell-permeable metabolic indicator dye. It appears blue in its oxidized form and is reduced to a red resorufin product (7-hydroxy-3H-phenoxazin-3-one) by NADH, NADPH, FADH, or other reductive species produced by the mitochondria citric acid cycle (Csepregi et al., 2018; Lavogina et al., 2022). Thus, cells that exhibit stronger redox reactions correspond to higher cellular metabolism (Wang et al., 2018). Other redox assays, such as MTT and XTT dyes, have been developed for the detection of cell metabolic activity (Morgan, 1998), including sperm cells (Berridge et al., 2005; Aziz, 2006). Compared to these oxidized-reduced reactions, the resazurin assay is not only time-efficient but also cost-effective (Sung et al., 2022). Indeed, the resazurin assay has been applied for the basic evaluation of sperm activity in boars (Zrimšek et al., 2004). However, to the best of our knowledge, the specific reaction conditions, including sperm concentration, semen volume, resazurin reagent volume, and reaction duration, have not been established. Furthermore, the correlation between the resazurin redox test and sperm quality parameters measured using CASA and FC remains unclear.

To enhance the utilization of the resazurin assay for evaluating boar sperm quality, our aim was to identify the optimal reaction conditions. Our combined data could optimize the conditions of the resazurin assay for boar semen and has also demonstrated the corresponding results of CASA and FC techniques. These methods may provide an alternative approach for assessing boar semen quality using basic equipment in a laboratory or animal field.

## Methods

### Animal management

All experiments were conducted in accordance with ethical treatment regulations and were approved by the Institutional Animal Care and Use Committee at the Livestock Research Institute (Authorization Number: N° TLRI-IACUC-111). Four adult Duroc-Meishan crossbred boars, aged 18 to 26 months, were selected as semen donors based on sperm tests, which included evaluating motility (> 80%) and viability (> 80%) over 5 consecutive semen collections. All animals were housed in a building with stable conditions, including controlled temperature ranging from 20-22°C and humidity ranging from 60-80%. They were received 7 pounds of feed daily containing 8.5 Mcal of metabolizable energy, 15.5% crude protein and 1.1% lysine and provided with water ad libitum at the Boar Breeding unit in Taiwan Livestock Research Institute (TLRI). The animals were under the surveillance and guidance of a certified veterinarian.

### Semen collection and preparation of simulated sperm samples

Boar semen was collected twice a week using the hand glove technique and stored in water-jacketed bottles (Trzcińska and Bryła, 2021). Immediately after collection, individual ejaculates from four boars were gently mixed, and the sperm concentration was determined using a photometer (SpermCue, Minitube) (Schäfer-Somi and Aurich, 2007). Semen samples were then simulated to represent different sperm qualities and prepared as previously described (Chen et al., 2020). In brief, the freshly collected semen was divided into two parts in order to prepare intact and damaged aliquots. The intact portion was kept at room temperature, while the damaged one was submerged in liquid nitrogen and then thawed at 37°C for two cycles in order to intentionally disrupt sperm characteristics. Different proportions of intact and damaged sperm were mixed to the designed ratios (intact/damaged) of 0/10, 2/8, 4/6, 6/4, 8/2, and 10/0 (v/v), representing various levels of sperm quality from low to high at 0, 20, 40, 60, 80, and 100%, respectively. Each tube of simulated sperm samples was then subjected to sperm quality analyses (Lin et al., 2019), including sperm motility using a computer-assisted sperm analysis system (CASA, CEROS II™, IMV Technologies), and sperm functional parameters such as viability, acrosome integrity, and mitochondrial activity using microcapillary flow cytometry (FC, Guava^®^ easyCyte 5HT, IMV Technologies).

### Dilution of simulated sperm samples

The average sperm concentration of boar semen is between 200 and 300 × 10^6^ cells/mL (Namuncura et al., 2020). Therefore, semen samples were prepared at concentrations of 300 and 150 × 10^6^ cells/mL to develop a new condition of resazurin reduction assay, which can be used in practical pig farms in the future. Briefly, the intact and damaged semen aliquots were prepared as previously described and serially diluted to 300 and 150 × 10^6^ cells/mL with phosphate-buffered saline (PBS) (Lonza #17-516F). After dilution, semen samples were mixed following the methodology described in the previous paragraph to generate simulated semen samples with varying levels of sperm quality. Subsequently, each sperm suspension was assessed using the resazurin assay, as well as CASA tests for sperm motility and FC tests for sperm functional parameters.

### Resazurin/Resorufin reduction assay

Resazurin, which is the active ingredient of the PrestoBlue™ reagent, was used here as a colorimetric method, following the instructions provided by the manufacturer (Invitrogen™ #A13261). PrestoBlue™ reagent, which is blue in color, is a non-toxic and cell-permeable compound. After entering live cells in the cellular reducing environment, resazurin is reduced to resorufin, resulting in a noticeable change in color to red. This change in color increases as the cellular metabolic activity increases. A dose-dependent assessment was conducted to determine the optimal reaction conditions of the resazurin assay in boar sperm samples. The assessment included different ratios of semen to PrestoBlue™ reagent (100:10, 200:10, and 200:20) (v:v, µL), as well as a time-dependent examination from 0 to 60 min with a 10-min interval. For each sample, three technical replicates of 100 or 200 µL of semen were transferred into a 96-well flat-bottom clear microplate (Greiner Bio-One). Then, 10 or 20 µL of PrestoBlue™ solution was added to each well. Immediately after addition, the microplate containing sperm and PrestoBlue™ mixtures was placed in a spectrophotometer (BioTek). The absorbance was then measured at 570 nm, with 600 nm being used as the reference wavelength. The microplate was incubated at 37°C for durations of 10, 20, 30, 40, 50, and 60 min, respectively, before measuring the absorbance. In addition, the semen suspensions were also evaluated for sperm motility and functional parameters. Subsequently, a correlation analysis was performed using the results of the resazurin/resorufin reduction rate to identify the conditions with which the sperm parameters were most strongly correlated, using a linear regression test.

### Sperm motility evaluation using CASA

Sperm motility was examined using CASA, as previously described (Lin et al., 2019). Sperm concentration was adjusted to 30 × 10^6^ cells/mL using PBS. A 2.5 μL aliquot was then loaded into a four-chamber counting slide (CellVision Technologies) and placed on a temperature-controlled stage at 37°C. Images were acquired from five fields of a slide chamber at a rate of 60 frames per second, resulting in a total of 30 frames. The threshold values of sperm parameters were defined based on the default settings from the company's installation and analyzed average path velocity (VAP), straight line velocity (VSL), and straightness (STR = VSL/VAP). Sperm motility results were presented as the percentage of total and progressive motility. Total motility was defined as the percentage of sperm showing a VAP greater than 15 μm/s, while progressive motility was defined as the percentage of sperm showing a VAP greater than 50 μm/s with STR greater than 70% (Sellem et al., 2015).

### Sperm functional parameters evaluation using FC

Sperm viability, acrosome integrity, and mitochondrial activity were analyzed using FC based on a previous study (Sellem et al., 2015). The FC contains one blue laser (488 nm) and two photodiodes for detecting forward and side scatter. Sperm cell emission properties are measured using three photomultiplier tubes: green (525/30 nm), yellow (583/26 nm), and red (680/30 nm). For each sample, a total of 5,000 sperm cells were recorded for scatter and fluorescence properties. These properties were then calculated to determine the percentage of cell populations carrying different fluorescence signals (Sellem et al., 2015).

**Sperm Viability Test:** In this study, a ready-to-use EasyKit™ Viability and Concentration Kit (IMV Technologies #024708) was utilized, following the instructions provided by the manufacturer. The kit contains SyBr14 and propidium iodide (PI) stains, which have differential permeability to distinguish between live (membrane intact) and dead (membrane damaged) cells. Ready-to-use 96-well microplates were first filled with 200 μL/well of EasyBuffer (IMV Technologies # 022162). Then, 57,000 sperm cells were added into the wells. Sperm and kit reagent mixtures were then incubated at 37°C for 10 min in the dark before the fluorescence signals were read by the FC. The results were expressed as the percentage of live sperm, which is also referred to as sperm viability.
**Sperm Acrosome Integrity Test:** An EasyKit™ Acrosome Integrity and Viability Kit (IMV Technologies # 025293) was used to detect the acrosome status. This kit includes peanut agglutinin (PNA), Syto83 (a cell-permeant orange nucleic acid stain), and PI dye. These substances bind to the outer acrosomal membrane, live sperm, and dead sperm, respectively. The preparation of the 96-well microplate buffer, sperm addition, and cell acquisition for FC are the same as the viability test, except that this assay requires a 45-min staining incubation. The results were presented as the percentage of intact acrosomes within live sperm, which is also referred to as sperm acrosome integrity.
**Sperm Mitochondrial Activity Test:** The EasyKit™ Mitochondrial Activity Kit (IMV Technologies # 024864) was used to assess the functionality of sperm mitochondria. This kit contains a JC-1 dye, which is a green-fluorescent monomer that exhibits green fluorescence at low mitochondrial membrane potential and forms red-fluorescent aggregates at higher potential. Sperm and kit reagent suspensions were protected from light and incubated at 37°C for 30 min before recording the fluorescence signals using the FC. The results were expressed as the percentage of polarized mitochondria, which corresponds to the activity of sperm mitochondria.

### Statistical analysis

Statistical analyses were performed using GraphPad Prism version 10. The data from simulated sperm samples in different levels of sperm quality from low to high, and their corresponding sperm parameters (motility and functional parameters), were assumed to follow Gaussian distributions. Therefore, a Pearson correlation coefficient test was conducted to investigate the correlations between the preparation of simulated sperm samples and various sperm quality parameters, in order to validate their reliability. Subsequently, a Pearson correlation coefficient test was used to evaluate the correlations between the resazurin/resorufin reduction rate, sperm total motility, progressive motility, viability, acrosome integrity, and mitochondrial activity with the dose-dependent and time-dependent variables. This test was performed on two groups with sperm concentrations of 300 and 150 × 10^6^ cells/mL, respectively. Finally, we identified a reaction condition that exhibited the highest correlation between the reduction rate of resazurin/resorufin and sperm parameters. This condition required the shortest incubation time and achieved a correlation coefficient above 0.9 for each measurement. We selected this condition from the groups with sperm concentrations of 300 and 150 × 10^6^ cells/mL, respectively. We then conducted a simple linear regression test to derive equations for calculating each sperm parameter based on the absorbance values obtained from the resazurin assay.

## Results

### Simulated semen samples highly correlate with sperm quality parameters

Sperm samples were prepared with the intended ratios of intact and damaged sperm aliquots to simulate semen with different levels of sperm quality: 0, 20, 40, 60, 80, and 100%. Sperm quality parameters of simulated semen suspensions, including total motility, progressive motility, viability, acrosome integrity, and mitochondrial activity, were all found to be highly correlated (r > 0.9) with the expected sperm quality (Table 1). This suggests that the methodology used to prepare the simulated semen was effective and reliable for the subsequent experiments.

**Table 1:** Boar sperm parameters obtained using computer-assisted sperm analysis (CASA) and flow cytometry (FC) at various sperm quality levels.

	**Sperm quality level (%)**	**0**	**20**	**40**	**60**	**80**	**100**		
CASA	Total motility	0	18.05	32.32	48.18	64.75	81.16	0.99	r
	Progressive motility	0	6.40	13.37	18.90	26.78	32.68	0.97	
FC	Viability	0.28	10.57	21.52	33.20	50.00	69.68	0.97	
	Acrosome integrity	0.12	9.68	18.23	30.70	45.07	61.30	0.98	
	Mitochondrial activity	5.48	17.18	30.77	43.98	59.95	72.72	0.99	

Sperm quality level refers to the proportion of intact sperm mixed with damaged sperm in a semen sample. Values are the means of 36 samples in 6 biological replicates. The letter “r” represents the correlation coefficient between sperm quality level, CASA, and FC parameters.

### Optimal reaction conditions for the resazurin/resorufin reduction assay

After a 20-min incubation of 100 μL of simulated semen (300 × 10^6^ sperm/mL) with 10 μL of resazurin reagent (Table 2), a strong correlation was observed (r > 0.9) between the rate of resazurin reduction and various sperm parameters, including total motility, progressive motility, viability, acrosome integrity, and mitochondrial activity. This reaction condition required the shortest reaction duration compared to the other conditions, even when using double the volume of semen (200 μL) or resazurin reagent (20 μL). For the samples of sperm concentration at 150 × 10^6^ cells/mL, the optimal condition was achieved by using 100 μL of simulated semen mixed with 10 μL of resazurin reagent and incubating for 40 min (Table 3).

**Table 2:** Pearson correlation coefficients were calculated between the resazurin reduction rate and boar sperm parameters at different time points, using different ratios of semen to PrestoBlue™ solution, with a sperm concentration of 300 x 10^6^ cells/mL.

			**Correlation coefficient (r)**		
		**Semen: PrestoBlue™ (v:v, µL)**	**100:10**	**200:10**	**200:20**
Sperm concentration = 300 x 10^6^ cells/mL	10 min	Total motility	0.905*	0.873*	0.804*
		Progressive motility	0.912*	0.876*	0.791*
		Viability	0.932*	0.941*	0.874*
		Acrosome integrity	0.920*	0.939*	0.871*
		Mitochondrial activity	0.845*	0.871*	0.802*
	20 min	Total motility	0.931*	0.908*	0.934*
		Progressive motility	0.933*	0.899*	0.922*
		Viability	0.955*	0.896*	0.901*
		Acrosome integrity	0.943*	0.899*	0.917*
		Mitochondrial activity	0.916*	0.933*	0.944*
	30 min	Total motility	0.952*	0.901*	0.881*
		Progressive motility	0.963*	0.903*	0.871*
		Viability	0.969*	0.848*	0.804*
		Acrosome integrity	0.972*	0.865*	0.825*
		Mitochondrial activity	0.921*	0.929*	0.909*
	40 min	Total motility	0.964*	0.898*	0.869*
		Progressive motility	0.972*	0.904*	0.870*
		Viability	0.966*	0.855*	0.807*
		Acrosome integrity	0.976*	0.866*	0.823*
		Mitochondrial activity	0.941*	0.929*	0.907*
	50 min	Total motility	0.965*	0.895*	0.914*
		Progressive motility	0.974*	0.897*	0.911*
		Viability	0.968*	0.846*	0.855*
		Acrosome integrity	0.978*	0.858*	0.871*
		Mitochondrial activity	0.940*	0.925*	0.943*
	60 min	Total motility	0.960*	0.882*	0.926*
		Progressive motility	0.970*	0.885*	0.922*
		Viability	0.958*	0.828*	0.865*
		Acrosome integrity	0.973*	0.840*	0.882*
		Mitochondrial activity	0.930*	0.912*	0.952*

Resazurin reduction rate refers to the total change in absorbance at 570 nm (using 600 nm as a reference wavelength) during various reaction durations (0, 10, 20, 30, 40, 50, and 60 min). Data was collected from 72 samples in 12 biological replicates. *The correlation is significant at the 0.01 level (two-tailed).

**Table 3:** Pearson correlation coefficients were calculated between the resazurin reduction rate and boar sperm parameters at different time points, using different ratios of semen to PrestoBlue™ solution, with a sperm concentration of 150 x 10^6^ cells/mL.

			**Correlation coefficient (r)**		
		**Semen: PrestoBlue™ (v:v, µL)**	**100:10**	**200:10**	**200:20**
Sperm concentration = 150 x 10^6^ cells/mL	10 min	Total motility	-0.311	0.815*	0.872*
		Progressive motility	-0.271	0.800*	0.866*
		Viability	-0.263	0.830*	0.923*
		Acrosome integrity	-0.253	0.826*	0.920*
		Mitochondrial activity	-0.441	0.855*	0.905*
	20 min	Total motility	0.656*	0.718*	0.846*
		Progressive motility	0.687*	0.678*	0.820*
		Viability	0.718*	0.771*	0.905*
		Acrosome integrity	0.727*	0.754*	0.896*
		Mitochondrial activity	0.528*	0.765*	0.876*
	30 min	Total motility	0.792*	0.875*	0.911*
		Progressive motility	0.808*	0.851*	0.888*
		Viability	0.834*	0.920*	0.950*
		Acrosome integrity	0.836*	0.911*	0.946*
		Mitochondrial activity	0.689*	0.902*	0.946*
	40 min	Total motility	0.972*	0.888*	0.931*
		Progressive motility	0.980*	0.898*	0.914*
		Viability	0.962*	0.915*	0.966*
		Acrosome integrity	0.966*	0.919*	0.963*
		Mitochondrial activity	0.965*	0.817*	0.953*
	50 min	Total motility	0.921*	0.947*	0.955*
		Progressive motility	0.921*	0.925*	0.952*
		Viability	0.935*	0.971*	0.972*
		Acrosome integrity	0.939*	0.966*	0.975*
		Mitochondrial activity	0.866*	0.972*	0.977*
	60 min	Total motility	0.916*	0.963*	0.963*
		Progressive motility	0.916*	0.948*	0.961*
		Viability	0.927*	0.978*	0.977*
		Acrosome integrity	0.934*	0.977*	0.979*
		Mitochondrial activity	0.865*	0.985*	0.985*

Resazurin reduction rate refers to the total change in absorbance at 570 nm (using 600 nm as a reference wavelength) during various reaction durations (0, 10, 20, 30, 40, 50, and 60 min). Data was collected from 72 samples in 12 biological replicates. *The correlation is significant at the 0.01 level (two-tailed).

### Equations for calculating sperm quality parameters from absorbance values

**Figure 1:**
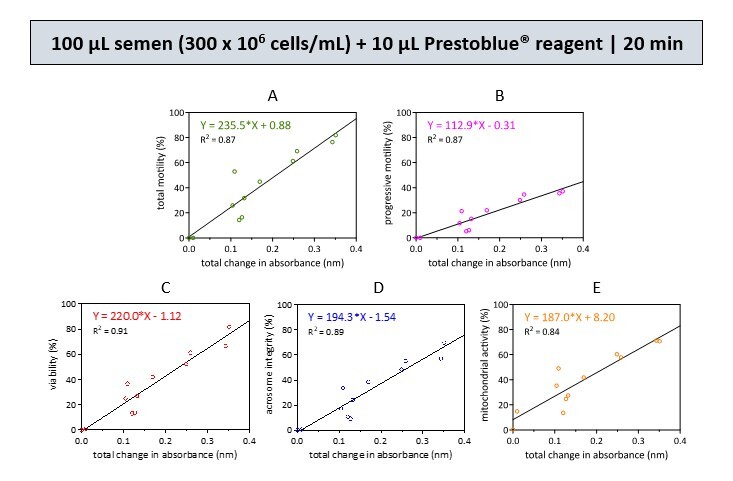
Linear regression analyses were conducted to examine the relationship between the resazurin reduction rate and various sperm parameters, including total motility (A), progressive motility (B), viability (C), acrosome integrity (D), and mitochondrial activity (E). An equation was created using the coefficients obtained from the best multiple regression analysis. This analysis was performed on a semen sample of 100 µL with a concentration of 300 x 10^6^ cells/mL. The sample was then incubated with 10 µL of Prestoblue® reagent for 20 min. These graphs demonstrate the goodness of fit (R^2^) of our data in the prediction equations based on the multiple regression analyses. Data was collected from 12 samples in 2 biological replicates.

**Figure 2:**
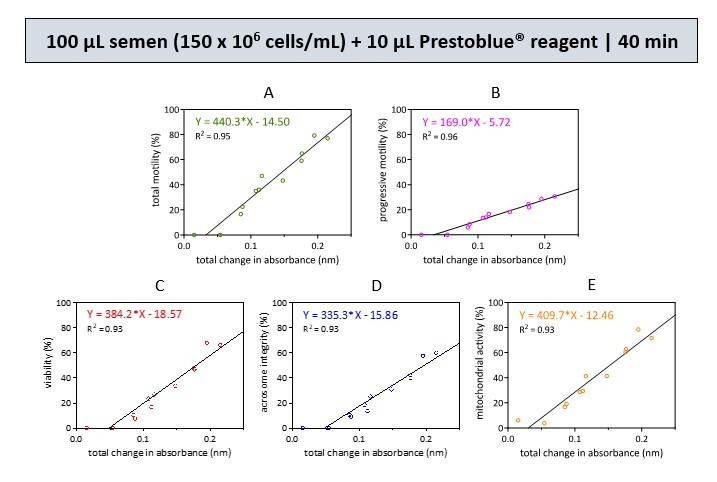
Linear regression analyses were conducted to examine the relationship between the resazurin reduction rate and various sperm parameters, including total motility (A), progressive motility (B), viability (C), acrosome integrity (D), and mitochondrial activity (E). An equation was created using the coefficients obtained from the best multiple regression analysis. This analysis was performed on a semen sample of 100 µL with a concentration of 150 x 10^6^ cells/mL. The sample was then incubated with 10 µL of Prestoblue® reagent for 40 min. These graphs demonstrate the goodness of fit (R^2^) of our data in the prediction equations based on the multiple regression analyses. Data was collected from 12 samples in 2 biological replicates.

A single optimal reaction condition was selected from simulated semen samples containing 300 and 150 × 10^6^ cells/mL, respectively. The results were then subjected to a simple linear regression test to obtain five equations, as indicated in the graphs (Figure 1 and 2). These equations can be used to calculate sperm quality parameters (Y) based on the corresponding changes in resazurin reduction absorbance (X).

## Discussion

A simple and rapid method for screening boar fertilizing capacity and assessing the quality of semen before insemination in the field is necessary and valuable. Thus, we have developed a new protocol based on the use of a resazurin redox dye in this study. To improve the use of the resazurin assay in evaluating boar sperm quality, we first created simulated semen samples with different levels of sperm quality (0, 20, 40, 60, 80, 100%) at concentrations of 300 and 150 × 10^6^ cells/mL. These concentrations are within the average range of sperm concentration found in semen ejaculate (Namuncura et al., 2020). Subsequently, the simulated semen was used to establish an optimal standard protocol based on the results of the resazurin colorimetric assay. Finally, the condition that showed the strongest correlation between resazurin redox rate and sperm parameters was selected to perform a linear regression test. This test aimed to derive five equations for calculating sperm total motility, progressive motility, viability, acrosome integrity, and mitochondrial activity based on the results of the resazurin assay. This protocol involves optimizing the reaction conditions and establishing a set of equations to determine various parameters related to sperm quality.

Indeed, the resazurin assay has been used to assess sperm quality in various species, including humans (Glass et al., 1991; Reddy et al., 1998), boars (Zrimšek et al., 2004), bulls (Dart et al., 1994), canines (Strzeżek et al., 2013), and rams (Wang et al., 1998), in previous studies. However, in these studies, the protocols for the resazurin assay did not specify a particular sperm concentration for the resazurin redox reaction, except for the study involving ram sperm. Based on the results of the present study, it was found that sperm concentration influenced the rate of resazurin reduction, leading to variations in colorimetric presence. Therefore, it is necessary to perform the resazurin assay with a consistent sperm concentration in order to ensure a reliable reaction. In addition, the study of ram semen (Wang et al., 1998) used a sperm concentration of 50 × 10^6^ cells/mL in the reaction. With such conditions, it took 60 min for the entire redox reaction to occur, suggesting that a more efficient condition was revealed in our study with semen at a concentration of 300 or 150 × 10^6^ sperm/mL.

Here, we defined two sets of reaction conditions for semen containing either 300 or 150 × 10^6^ sperm cells/mL. Indeed, the reaction duration is 40 min when the sperm concentration is 150 × 10^6^ cells/mL, which is double the duration of 300 × 10^6^ cells/mL of semen. Despite being less time-efficient, this set of conditions is necessary to be included according to the regular sperm concentration in boars (Namuncura et al., 2020). Increasing the volume or concentration of the resazurin reagent may reduce the reaction duration, making it more time-efficient. However, this would also lead to a lower cost-efficiency due to the consumption of twice the volume of reagent.

We have shown here that the resazurin reduction activity is highly correlated with all sperm quality parameters after moderate incubation periods. The occurrence of resazurin reduction depends on mitochondrial activity, which is also related to sperm motility and viability (Marchetti et al., 2002; Alamo et al., 2020). Therefore, it is reasonable to observe a significant correlation between the resazurin reaction, sperm motility, viability, and mitochondrial activity. Remarkably, a high correlation was also found between the resazurin reaction and acrosome integrity, which has never been reported to be associated with cellular reducing activity but rather with mitochondrial membrane potential. This potential is an important parameter that reflects mitochondrial function (Gallon et al., 2006). These facts suggest an indirect correlation between the resazurin redox reaction and acrosome status, rather than a direct correlation.

It is obvious that semen dilution methods, the various compositions of extenders, and the storage temperature are known to influence semen quality (Vyt et al., 2004; López Rodríguez et al., 2012; Rodriguez et al., 2017; Henning et al., 2022). In this study, semen samples were diluted with PBS before being mixed as simulated semen. This dilution could be expected to result in a significant decrease in sperm quality parameters with an increase in incubation at 37°C (Sa et al., 2013; Quirino et al., 2023). However, the purpose of this study was to define an alternative protocol for testing sperm quality instead of CASA or FC. The sperm quality parameters were measured just after collection and before dilution. It is necessary to incubate tested semen samples at 37°C for the Resazurin/Resorufin Reduction requirement (Aziz, 2006). Therefore, it might be worthwhile to modify the defined protocols by using a semen extender to preserve boar sperm functionalities. However, it is necessary to consider whether the compositions of the extender disrupt or change the Resazurin/Resorufin reduction process.

It has been reported that the seminal biochemical background could influence the resazurin reduction test (Zalata et al., 1998), which may require an additional removal process of seminal plasma to avoid such disturbance (Strzeżek et al., 2013). The current study aimed to develop a fast and simple test for evaluating boar semen quality before artificial insemination in practice. Thus, an additional centrifugation for semen samples could result in a significant increase in workload for practical application. This underscores the necessity of comparing the differences between seminal plasma and PBS when conducting the resazurin reduction test for the next stage of development. This data could be used as background compensation to enhance the reliability and predictability of the defined protocol.

This study provides the first information on the optimized conditions for performing the resazurin redox dye assay to assess sperm quality in boars. Despite the fact that sperm morphology and biological characteristics may vary among animal species, we believe that this colorimetric assay, based on dehydrogenase reduction, could be widely adapted in different species. This is because sperm cells share the same mechanisms of mitochondria dehydrogenase. However, delicate modifications and careful validations may be required before applying them to animal species other than boars.

Using conventional microscopy to evaluate sperm motility and morphology is an efficient strategy to assess the boar's fertilizing capacity (Tanga et al., 2021). Due to the low cost of a basic microscope, it can be affordable in most spermatology laboratories and pig farms. However, it requires complex and lengthy working schedules to compare each individual sample. The advantage of our new resazurin redox protocol includes efficient handling capacity and low equipment costs. It is possible to analyze 96 reactions in a microplate reader simultaneously, and the cost of acquiring a spectrophotometer is lower than that of CASA or FC.

## Conclusion

To conclude, we optimized the reaction conditions of the resazurin assay used to assess the quality of boar semen at two different sperm concentrations. In addition, we defined five regression equations used to obtain sperm parameters, including total motility, progressive motility, viability, acrosome integrity, and mitochondrial activity. These parameters were calculated based on the absorbance of the resazurin redox reaction. The resazurin assay can be performed with multiple samples simultaneously in a 96-well microplate, using only a plate reading spectrophotometer to interpret the data instead of expensive and complex devices. All of these advantages support this assay as a simple, rapid, and efficient method that can be applied in a laboratory or animal field using basic equipment.
